# Consanguineous marriages and endemic malaria: can inbreeding increase population fitness?

**DOI:** 10.1186/1475-2875-7-150

**Published:** 2008-08-02

**Authors:** Srdjan Denic, Nicolas Nagelkerke, Mukesh M Agarwal

**Affiliations:** 1 Department of Internal Medicine, Faculty of Medicine and Health Sciences, UAE University, PO Box 17666, Al Ain, Abu Dhabi, UAE; 2 Department of Community Medicine, Faculty of Medicine and Health Sciences, UAE University, PO Box 17666, Al Ain, Abu Dhabi, UAE; 3 Department of Pathology, Faculty of Medicine and Health Sciences, UAE University, PO Box 17666, Al Ain, Abu Dhabi, UAE; 4 Department of Medical Microbiology and Infectious Diseases, University of Manitoba, 730 William Avenue, MB, R3E 0W3,Winnipeg, Canada

## Abstract

**Background:**

The practice of consanguineous marriages is widespread in countries with endemic malaria. In these regions, consanguinity increases the prevalence of α^+^-thalassemia, which is protective against malaria. However, it also causes an excessive mortality amongst the offspring due to an increase in homozygosis of recessive lethal alleles. The aim of this study was to explore the overall effects of inbreeding on the fitness of a population infested with malaria.

**Methods:**

In a stochastic computer model of population growth, the sizes of inbred and outbred populations were compared. The model has been previously validated producing results for inbred populations that have agreed with analytical predictions. Survival likelihoods for different α^+^-thalassemia genotypes were obtained from the odds of severe forms of disease from a field study. Survivals were further estimated for different values of mortality from malaria.

**Results:**

Inbreeding increases the frequency of α^+^-thalassemia allele and the loss of life due to homozygosis of recessive lethal alleles; both are proportional to the coefficient of inbreeding and the frequency of alleles in population. Inbreeding-mediated decrease in mortality from malaria (produced via enhanced α^+^-thalassemia frequency) mitigates inbreeding-related increases in fatality (produced via increased homozygosity of recessive lethals). When the death rate due to malaria is high, the net effect of inbreeding is a reduction in the overall mortality of the population.

**Conclusion:**

Consanguineous marriages may increase the overall fitness of populations with endemic malaria.

## Background

Marriages between close biological relatives account for up to 60% of all marriages in many parts of Asia, Middle East and Africa [1]. A common finding among consanguineous populations is their long history of exposure to malaria. In fact, the frequency and degree of consanguineous marriages correlates with the geographic distribution and intensity of *Plasmodium falciparum *in the population [2]. Today, α^+^-thalassemia has become the most common monogenic disorder in humans potentially because it decreases the probability of death from infection with *P. falciparum *[3-5]. An earlier study has shown that the selection of many recessive alleles can be accelerated by inbreeding [6] and, recently, this has been demonstrated for α^+^-thalassemia in regions where malaria is endemic [7].

The widespread practice of consanguineous marriages has conventionally been attributed to its multiple social benefits, e.g., the aggregation of economic wealth, the better treatment of spouse, and an increased family stability and security [1,8,9]. However, this theory of social benefits as being the main motivation for consanguineous marriages is unconvincing because the same benefits would also accrue in other populations, should they have chosen to be consanguineous. Moreover, consanguinity is found in societies within the same geographic area despite being racially, linguistically, religiously, and historically very heterogeneous [1,2]. It seems unlikely that such a cultural trait, which lowers population fitness, has spread just because of its socio-economic usefulness amongst these very diverse populations. In this study, we examined the potential positive effects of inbreeding (through selection of α^+^-thalassemia) versus its well established harmful consequences.

## Methods

### The model

The genetic benefits (through α^+^-thalassemia allele) of inbreeding against the biological costs (via recessive lethal alleles) were evaluated in a stochastic model. The model has been verified by producing the results predicted by analytical methods (detailed in [7]) and uses the odds of survival of different α^+^-thalassemia genotypes from a field study [3]. In brief, the consanguineous and non-consanguineous populations were allowed to grow; their size (relative fitness) and α^+^-thalassemia allele frequency were compared. An initially large population (*n *= 1000) comprising of αα/αα genotypes was randomly seeded with α^+^-thalassemia using -α/αα genotype, so that the initial allele frequency was 0.03. The model was restricted to exclusively "large" populations as the effect of inbreeding on the selection of recessive and codominant alleles is significantly less in smaller populations [6,7]. Additionally, when malaria emerged as an epidemic infection 4,000 to 10,000 years ago, the Agrarian revolution had already caused a population explosion, an epidemiological pre-requisite for the appearance of malaria as an epidemic infection [10,11]. In this model, the population grows with the mating of a randomly chosen pair of individuals with a predetermined mean number of offspring; child's genotype is assigned using Mendelian rules of inheritance. After the mean coefficient of inbreeding (*F*) was allocated to a population, the couple was made consanguineous with a probability that equals the mean coefficient of relatedness, R (*R *= 2*F*). Biological relatedness of the couple was tested for each of the two alleles and, if found to be absent, another unmarried individual from the population was chosen and tested; this was continued until a biologically related individual was found -when a new marriage was arranged [7]. As only the surviving offspring become members of the next generation, there is no overlap between generations. In human consanguineous populations, highest reported *F *is 0.045 but, in the simulated experiment, the range was extended up to 0.09 because historically higher rates of inbreeding are possible [12,13].

**Figure 1:**
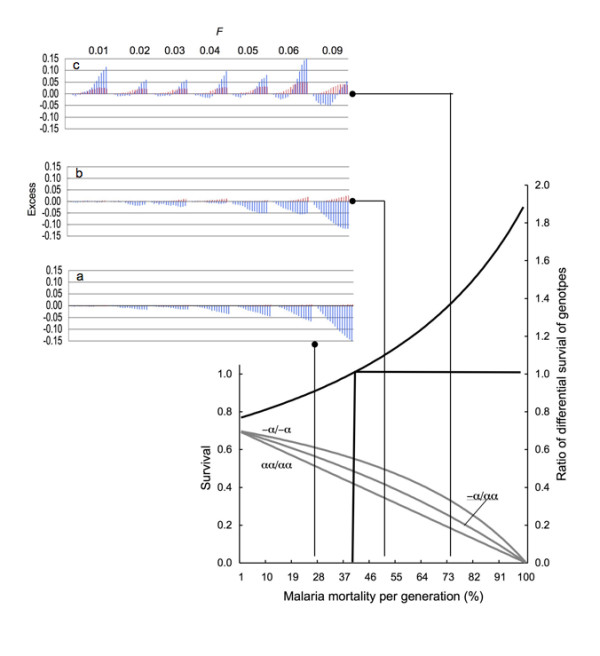
**The relative excess of α^+^-thalassemia allele frequency (red bars) and population size (blue bars) per generation in an inbred population when compared with a non-inbred population.** The survivals (*S*) of the three genotypes used in this simulation are shown in the lower part of graph at the intersections with vertical lines which point to the results in the upper part of graph. The ratio of differential survival of genotypes = (*S*_-α/-α _- *S*_-α/αα_)/(*S*_-α/αα _- *S*_αα/αα_).

Mortality from *P. falciparum *is highest in the first five years of life; it decreases with subsequent infections and, during a single epidemic, malaria can kill up to 50% of a susceptible population. Mortality rates of malaria differ between various geographic areas; in the same area they may vary over time [5]. In this simulation, mortality rate was per generation, which was made constant throughout time. The survival probabilities of α^+^-thalassemia genotypes were derived from the field study data obtained in a population from an area with endemic malaria. The odds ratio (OR) for the development of severe forms of the disease that precede death from malaria, is lower in heterozygotes (with a single alpha gene deletion, -α/αα genotype, OR = 0.66) and is further decreased in homozygotes (with two alpha gene deletions, -α/-α genotype, OR = 0.40) than in those without any α-thalassemia mutation (αα/αα genotype, OR = 1.0) [3]. The survival of each of the three genotypes depends on the mortality from malaria and is shown in Figure 1. Survival (*S*) for the three genotypes is derived from the odds of death from malaria (*r*), which are extrapolated to equal the odds of the severe form of the disease, and mortality rate from malaria (*p*) so that

*S *= 1 - *p'*

$$p'=\frac{rp}{\left(1-p+rp\right)}$$

In order to account for other causes of death, this result is scaled down to 0.7, i.e., 0.3 of all deaths are arbitrarily ascribed to non-malarial causes. The offspring of consanguineous families have a higher number of deaths (in the years prior to their reproduction) than offspring of non-consanguineous families; these deaths are due to homozygosity of harmful recessive alleles (inbreeding depression). An individual has on average of 1.4 recessive lethal alleles and the probability of excessive deaths due to inbreeding equals 0.7 *F *[13]. To account for this mortality in our model, all surviving children were exposed to an additional risk of 0.7*F *of dying before being allowed to reproduce; in the model, this consistently depressed population size in every generation by 0.7*F*.

In each set of simulations, the starting allele frequency is 0.03, *n *= 1000. The mean number of children per couple varied between 5 and 14 in order to allow a positive population growth and prevent extinction of population when malaria mortality is high. However, all comparisons of inbred and outbred populations were performed using the same set of parameters except the one which was being tested. All results are the means of 300 simulation runs.

### Calculation of relative fitness and allele frequency

**Figure 2:**
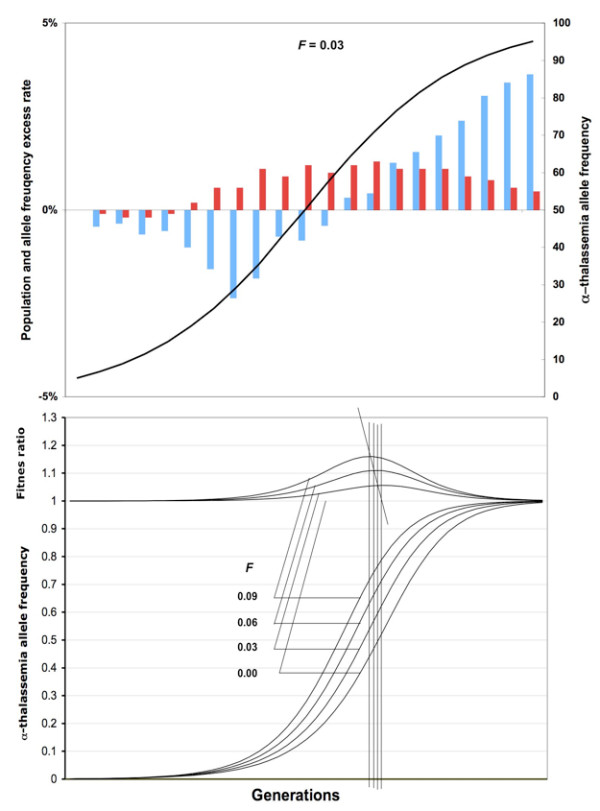
**Effect of α^+^- thalassemia frequency on the relative fitness of inbreeding populations in the stochastic (upper panel) and analytic model (lower panel).** In the upper panel, initial negative excess of relative fitness (blue bars) in the inbred population is the effect of recessive lethal alleles. An excess of α^+^-thalassemia allele (red bars) is seen after 5–6 generations – after allele frequency (black S-shape line) is increased to around 0.2. Relative excess of α^+^-thalassemia is maximal when its frequency is in the middle of the range (~0.35–0.7). Results are for *n *= 1000 and the ratio of differential survival = 1.39. In the lower panel, the calculated relative fitness includes only the effect of α^+^-thalassemia. The fitness ratio is the size of inbred population divided with that of outbred population. The results are for *n *→ ∞ and the ratio of differential survival = 1.39.

The relative fitness (*w*) and allele frequency change (Δ_s_*p*) in Figure 2 were determined from

$$\begin{array}{l} w=1-\left(1-F\right)\left(2pqsh+q^{2}s\right)-Fqs\\ \Delta_{s}p=\left(1-F\right)\frac{pqs\left[ph+q\left(1-h\right)\right]}{w}+F\frac{pqs}{w} \end{array}$$

*h *and *s *being 0.666 and 0.4, respectively, and corresponding to the ratio of differential survival of 1.39 and *n*→∞ in the stochastic model [7,14].

## Results and Discussion

When the mortality from malaria is low, consanguinity depresses the population with α^+^-thalassemia by causing an excessive number of deaths via recessive lethal alleles and by negligibly retarding the selection of α^+^-thalassemia allele (Figure 1a). The latter occurs when the difference between survival of -α/-α homozygote and -α/αα heterozygote genotypes is smaller than the difference between survival of -α/αα heterozygote and αα/αα homozygote (ratio of differential survival of genotypes < 1.0). This is also confirmed analytically, so, when *F *> 0 and

*S*_-α/-α _- *S*_-α/αα _<*S*_-α/αα _- *S*_αα/αα_,

the sum of all the three products of genotype frequencies [*q*^2^(1 - *F*) + *qF*, 2*pq*(1 - *F*) and *p*^2^(1 - *F*) + *pF*)] and their survival is always smaller than when *F *= 0 [14]; this also applies to all allele frequencies (*p *and *q *= 1 - *p*). With an increase in mortality due to malaria, the ratio of the differential survival increases to 1.0 (Figure 1, lower graph) at which point, inbreeding has neither a negative nor a positive effect on the speed of selection of α^+^-thalassemia- the inbreeding depression being solely due to the effect of lethal recessive alleles. When the ratio of differential survival of genotypes becomes > 1.0, inbreeding starts to accelerate the selection of α^+^-thalassemia. This causes an excess of α^+^-thalassemia frequency in the inbred population, which increases its relative fitness in comparison to an outbred population (Figure 1b and 1c). This gain in relative fitness partially or fully compensates inbreeding depression (halting the expansion of inbreeding depression) due to recessive lethal alleles as clearly illustrated by Figure1b.

As the death rate due to malaria increases, the relative excess of the frequency of α^+^-thalassemia in inbred populations increases further, and inbreeding depression may switch to inbreeding elevation, which takes place in populations with an *F *of 0.01 to 0.06 (Figure 1c), conspicuously within the range of *F *in consanguineous human populations (0 <*F *≤ 0.045) reported over the last half century [13,14].

**Figure 3:**
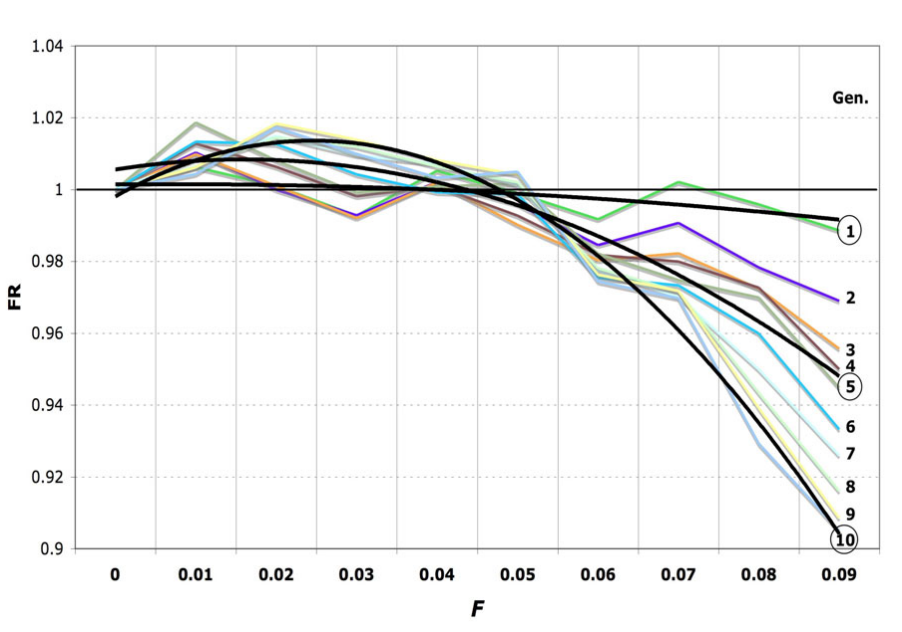
**Inbred/outbred fitness ratios (FR) of populations with a high α^+^-thalassemia frequency over ten generations. **At the start of simulation, the frequency of α^+^-thalassemia is 0.5. The black lines are trends for the first, fifth and tenth generation. FR > 1 is seen in every generation for all 0 <*F *< 0.05. This graph demonstrates robustness of inbreeding elevation found at all levels of inbreeding which were observed in human field studies, i.e., 0 <*F *≤ 0.045 [13,14].

When the initial frequency of α^+^-thalassemia is low (0.03), the onset of inbreeding elevation is delayed until the selection increases the frequency of α^+^-thalassemia (Figure 1b and 1c). The relative gain in fitness is greatest when the frequency of α^+^-thalassemia is around 0.5 (Figure 2); then the inbreeding elevation is observed immediately, from the first generation, and continuously thereafter in all populations with 0 <*F *< 0.05 (Figure 3). In native societies of the Arabian Peninsula, for example, α^+^-thalassemia allele frequency is around 0.3 and their mean *F *varies between 0.02 and 0.03, which suggests that overall consanguineous marriages have been genetically beneficial (before the control of malaria during the recent times) [15,16].

**Figure 4:**
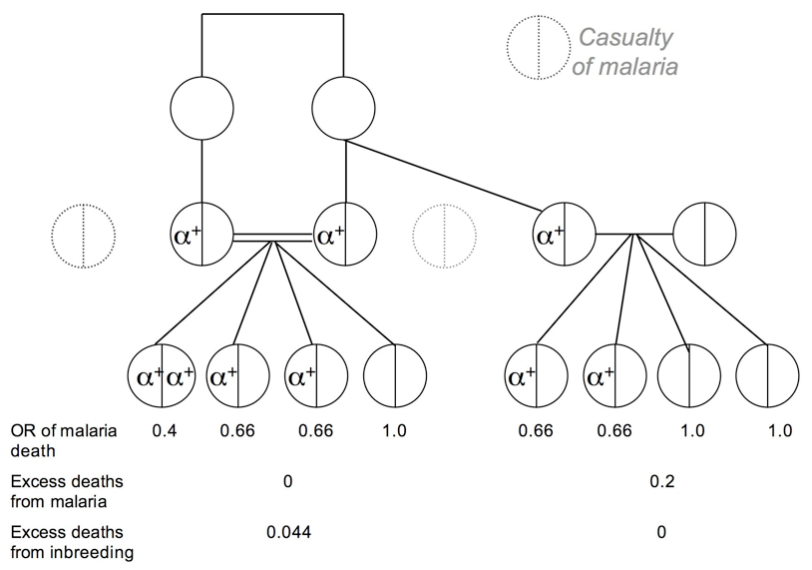
**Initiation of consanguineous marriages.** In family with α^+^-thalassemia, shortage of marriageable candidates leads to cousin-cousin and "cousin"-outsider unions. With the likely distribution of α^+^-thalassemia alleles shown above, on an average, 20% more children in this consanguineous union will survive malaria than from a non-consanguineous union. Here, the excess deaths due to inbreeding = 0.7*F*, i.e., 4.4% [13].

The reasons for the practice of consanguineous marriages to begin and persist in a population (with a low frequency of α^+^-thalassemia) after it experiences an inbreeding depression (Figure 1, upper graph) remain unclear. It is also uncertain why consanguinity should increase in populations at the time of increased mortality, e.g., cholera and famine epidemics [17,18]. Figure 4 shows how the benefits of cousin-cousin marriages are obvious but not its hazards. When there are a very few α^+^-thalassemia alleles in any population, a carrier family may opt for a cousin-cousin marriage because of a relative shortage of suitable marriage partners – caused by the high mortality due to malaria. In such consanguineous unions, both spouses are likely to be α^+^-thalassemia heterozygotes. Conversely, if another member of the same family (who is likely to be a carrier), marries an outsider- the spouse is likely to be a non-carrier. On an average, 20% more children from consanguineous unions would survive malaria (Figure 4) – a survival advantage sufficiently big to be noticed by members of a population under stress. Hence, more consanguineous marriages would be encouraged to take place among α^+^-thalassemia-carrier families. A support for unequal benefit to families from consanguineous marriages (illustrated in Fig. 4) is the observation that children of consanguineous parents are themselves more likely to enter into consanguineous unions than the children of non-consanguineous parents [19]. However, even in non-carrier families, consanguinity may not be discouraged despite its genetic dangers (like childhood deaths and increased congenital malformations). This is because for any specific morbidity to be noticed, the difference from the reference (non-consanguineous, in this case) has to be sufficiently higher than 5%, but is generally much below this threshold [1,13,20,21]. In addition, the excess deaths due to recessive lethal alleles may be masked by a high overall death rate. This mechanism for initiation of consanguinity and its persistence suggests that consanguinity can originate *de novo *rather than spread by acculturation. Further, the genetic benefits from inbreeding may strengthen the development of endogamy and tribalism. This to some extent corroborates findings from India which has over 50,000 brotherhoods and the frequency of α^+^-thalassemia is higher in tribal than in city populations [4,22]. As the frequency of α^+^-thalassemia increases over time, more families would experience the net genetic benefits of consanguinity. This could explain the social permissiveness towards consanguinity in regions where it is beneficial, e.g., in the societies from regions with endemic malaria [23].

The results presented agree with the historic conditions, which existed in the early human settlements, after the Agrarian revolution. At that time, populations increased rapidly due to better availability of food through farming and animal herding. However, the crowding, poor hygiene and proximity to animals contributed to the potential emergence of malaria and other epidemic infections [10,11]. Thus, when human survival became adversely affected by malaria, intra-family unions resulted in better survival of the offspring. A recent report of inbred families having more children than less inbred families in populations that never experienced malaria [24], further supports a role of human inbreeding as a facilitator of adaptation. In our globalized world with greater than ever mixing of populations, diseases like tuberculosis and AIDS are still the leading causes of death; protection against both is provided by codominant and recessive alleles [25,26] whose selection could be accelerated by inbreeding.

## Conclusion

Human inbreeding enhances the speed of fixation of recessive and codominant alleles. Consequently, the elimination of recessive lethal alleles is increased by an excessive mortality of children in consanguineous populations. However, an enhanced speed of selection of the codominant α^+^-thalassemia allele (in such inbred populations) increases the relative fitness against malaria. When mortality from malaria is high, this increase in fitness could offset the loss of life resulting from inbreeding. Therefore, consanguinity augments the fitness of a population with endemic malaria through its effect on α^+^-thalassemia allele.

## Competing interests

The authors declare that they have no competing interests.

## Authors' contributions

SD designed and obtained funds for the study, conducted the experiment, analyzed results and drafted the manuscript. NN analyzed results, provided analytical conformation of experimental results, and revised intellectual content of manuscript. MMA revised intellectual content and redrafted the manuscript. All authors contributed to writing and read and approved the final manuscript.
